# Lung tissue phantom mimicking pulmonary optical properties, relative humidity, and temperature: a tool to analyze the changes in oxygen gas absorption for different inflated volumes

**DOI:** 10.1117/1.JBO.27.7.074707

**Published:** 2021-11-01

**Authors:** Andrea Pacheco, Konstantin Grygoryev, Walter Messina, Stefan Andersson-Engels

**Affiliations:** a Biophotonics@Tyndall, IPIC, Tyndall National Institute, Lee Maltings, Dyke Parade, Cork, Ireland; b University College Cork, Department of Physics, Cork, Ireland

**Keywords:** lung phantom, gas spectroscopy, inflated volume, alveolar structure

## Abstract

**Significance:**

Gas in scattering media absorption spectroscopy (GASMAS) enables noninvasive gas sensing in the body. It is developing as a tool for diagnosis and monitoring of respiratory conditions in neonates. Phantom models with relevant features to the clinical translation of GASMAS technology are necessary to understand technical challenges and potential applications of this technique. State-of-the-art phantoms designed for this purpose have focused on the optical properties and anthropomorphic geometry of the thorax, contributing to the source–detector placement, design, and optimization. Lung phantom mimicking the alveolar anatomy has not been included in the existent models due to the inherent complexity of the tissue. We present a simplified model that recreates inflated alveoli embedded in lung phantom.

**Aim:**

The goal of this study was to build a lung model with air-filled structures mimicking inflated alveoli surrounded by optical phantom with accurate optical properties ($\mu_{a}=0.50\;{\mathrm{cm}}^{-1}$ and $\mu_{s}^{\prime }=5.4\;{\mathrm{cm}}^{-1}$) and physiological parameters [37°C and 100% relative humidity (RH)], and to control the air volume within the phantom to demonstrate the feasibility of GASMAS in sensing changes in pulmonary air volume.

**Approach:**

The lung model was built using a capillary structure with analogous size to alveolar units. Part of the capillaries were filled with liquid lung optical phantom to recreate scattering and absorption, whereas empty capillaries mimicked air filled alveoli. The capillary array was placed inside a custom-made chamber that maintained pulmonary temperature and RH. The geometry of the chamber permitted the placement of the laser head and detector of a GASMAS bench top system (MicroLab Dual $O_{2}/H_{2}O$), to test the changes in volume of the lung model in transmittance geometry.

**Results:**

The lung tissue model with air volume range from $6.89\times 10^{-7}\;m^{3}$ to $1.80\times 10^{-3}\;m^{3}$ was built. Two measurement sets, with 10 different capillary configurations each, were arranged to increase or decrease progressively (in steps of $3.93\times 10^{-8}\;m^{3}$) the air volume in the lung model. The respective GASMAS data acquisition was performed for both data sets. The maximum absorption signal was obtained for configurations with the highest number of air-filled capillaries and decreased progressively when the air spaces were replaced by capillaries filled with liquid optical phantom. Further studies are necessary to define the minimum and maximum volume of air that can be measured with GASMAS-based devices for different source–detector geometries.

**Conclusions:**

The optical properties and the structure of tissue from the respiratory zone have been modeled using a simplified capillary array immersed in a controlled environment chamber at pulmonary temperature and RH. The feasibility of measuring volume changes with GASMAS technique has been proven, stating a new possible application of GASMAS technology in respiratory treatment and diagnostics.

## 1 Introduction

The translation of light-based technologies into the clinic has been growing over the past decades. Biophotonics tools are starting to account for a larger proportion of next-generation diagnostic and therapeutic modalities, given that they provide information of tissue functionality and composition with minimal invasiveness. One of the major challenges in advancing biophotonics-based medical devices is transforming signals into useful information that can assist the clinicians in the treatment and diagnosis of diseases.^1^ In this work, we contribute to the understanding of a potential application of an existent light-based technology for localized air volume assessment in lung tissue.

### 1.1 Respiratory Physiology and Clinical Relevance of Pulmonary Volumes

**Fig. 1:**
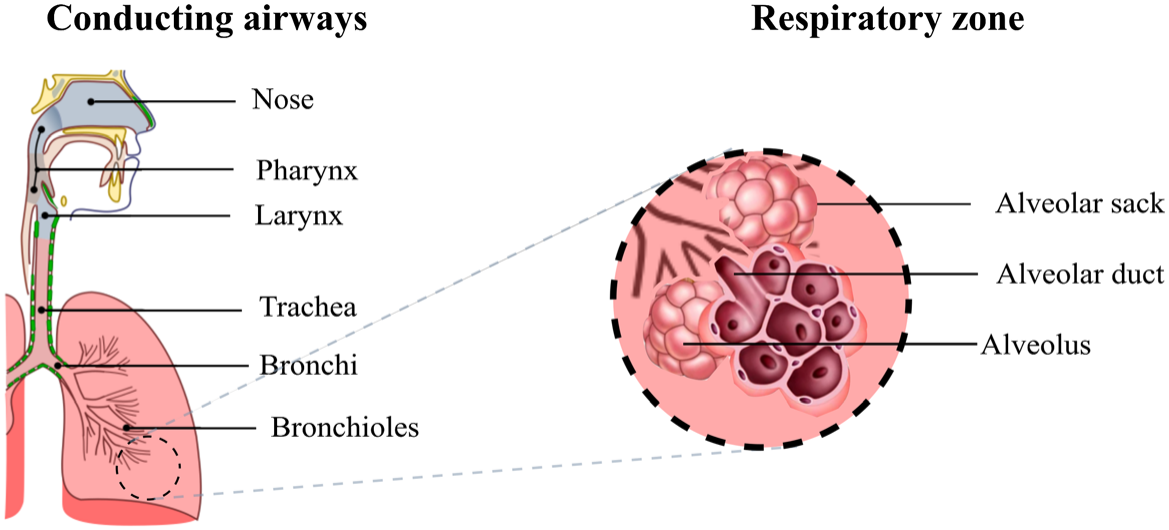
Diagram of the respiratory airways. The conducting airways warm up and moisten the inhaled air, the gas exchange occurs in the respiratory zone where the alveoli are located.

The respiratory system consists of conducting and respiratory airways (Fig. 1). The conducting airways lack alveoli and take no part in gas exchange. Their main function is to conduct, clean, heat up, and humidify the air to 37°C and 100% relative humidity (RH) as it reaches the alveolar sacks.^2^ The respiratory airways make up most of the lung and is the region where gas exchange occurs, and it is composed by millions of alveoli (∼0.3  mm in diameter each).^3^

The pulmonary gas volume is constantly changing during respiration. The four standard volumes corresponding to different degrees of inhalation or exhalation are classified as: tidal volume (amount of gas that can be inhaled or exhaled during a respiratory cycle: normal breathing); inspiratory reserve volume (the maximum amount of gas that can be inhaled beyond the normal tidal volume: deep breathing); expiratory reserve volume (volume of gas that can be exhaled by force beyond exhalation of the normal tidal volume); and residual volume (volume of gas that remains in the lung after maximal expiration). To assess and diagnose obstructive and restrictive pathologies, the measurement of respiratory volumes is of clinical importance.^4^

### 1.2 Gas in Scattering Media Absorption Spectroscopy

Gas in scattering media absorption spectroscopy (GASMAS) is a gas spectroscopy technique that uses tunable diode laser spectroscopy to measure the concentration of gases enclosed by diffuse media in a noninvasive way.^5^ The clinical applications of GASMAS include: assessment of infection in paranasal sinuses,^6^ diagnosis of otitis,^7^ early diagnosis of osteonecrosis,^8^ and monitoring of oxygen ($O_{2}$) and water vapor ($H_{2}O$) in the lungs of neonates.^9^^–^^12^ The studies advancing the clinical translation of GASMAS into respiratory healthcare have focused on neonates as the thickness of protective organs surrounding their lungs is within the limits of penetration depth for near-infrared light.^13^^,^^14^

GASMAS exploits the fact that the absorption signal of gases is unique against the tissue background. The absorption bands of $O_{2}$ and $H_{2}O$ are typically 0.001 nm,^15^ which can be distinguished from the absorption imprint of the organs around the lungs (both liquid and solid), which is generally in the 10 nm range.^16^ In clinical studies, the thoracic walls are illuminated with a diffuse source, and the scattered light reaching a photodetector is used to identify the absorption signal of molecular $O_{2}$, by means of the Beer–Lambert law equation, which states that the concentration of the gas c can be calculated from the absorbance (logarithm of the ratio of the source light intensity $I_{0}$ to detected intensity  I) when the molar absorption [ε(λ)] and the gas absorption path length (l)^17^ are known:

$$I=I_{0}e^{-\varepsilon cl}.$$ (1)

In the case of gas assessment in the lung, the thorax is illuminated with two wavelengths chosen to minimize the absorption by tissue:^12^ tunable diode lasers operating at 760 nm are typically selected to match the absorption bands of $O_{2}$, and 820 or 935 nm to match absorption of $H_{2}O$. The last is used as reference gas. Since temperature and RH of the lungs are well known, the concentration of $H_{2}O$ is calculated using the ideal gas law, with the partial pressure computed by means of the Arden Buck equation, which states that the partial pressure of vapor saturation in moist air ($e_{w}^{\prime }$) at ambient temperature T and pressure P is given as^18^

$$e_{w}^{\prime }=[1.0007+(3.46\times 10^{-6}P)]\times 6.1121\;\exp[\frac{17.502\;T}{240.97\;+\;T}].$$ (2)

The Beer–Lambert law is applied to the $H_{2}O$ absorption spectra to estimate l inside the lung. Consequently, the calculated value of l is input into Eq. (1) together with the absorption signal at 760 nm to estimate the $O_{2}$ concentration. This gas absorption path length approximation is valid for wavelengths that are spectrally close and interacting with identical media.^19^

The lung volume assessment is currently done using spirometry, flow volume curves, and/or plethysmography. However, these tests do not provide information of localized volume changes and patients are asked to make inspiratory and expiratory manoeuvres for volume assessment to be performed.^20^ This represents a limitation in measuring the pulmonary volumes in neonates or patients under anesthesia. GASMAS can potentially solve such limitations given that the light absorbance depends on l, which is directly proportional to gas volume.

In this study, we prove the feasibility of measuring changes in gas volume using GASMAS technology. The absorption imprint of molecular $O_{2}$ was acquired using a pulmonary phantom model that includes air pockets mimicking the size, temperature, and RH of alveoli, surrounded by liquid phantom matching absorption and scattering of lung tissue.

## 2 Methods

### 2.1 Construction of Lung Tissue Phantom

The phantoms previously created to investigate the clinical application of GASMAS in neonatal respiratory healthcare had a hollow cavity with the outer geometry of the lung without the inner structure mimicking the alveolar anatomy.^21^^,^^22^ The phantom designed in this study recreated inflated alveoli surrounded by lung tissue.

The simplified pulmonary model required a three-dimensional (3D) rectangular holder (0.025×0.014×0.035  m), printed with clear resin (RS-F2-GPCL-04 Formlabs, Form 2) filled with a set of 229 glass capillaries (maximum amount of capillaries that fit in the holder forming a gridded array) of $0.25\times 10^{-3}\;m$ inner radius and $125\times 10^{-3}\;m$ length (SIGMA, batch Z114952). A fraction of the capillaries was filled with air (mimicking the alveoli) and the remaining capillaries were filled with liquid optical phantom of lung tissue ($\mu_{a}=0.50\;{\mathrm{cm}}^{-1}$ and $\mu_{s}^{\prime }=5.4\;{\mathrm{cm}}^{-1}$, see Sec. 2.1).^16^ The number of capillaries filled with liquid phantom was altered and distributed randomly to reduce or increase the amount of air in the probed volume. To perform the measurements, the capillary array was enclosed in a controlled environment chamber (designed to fit the source and detector of a GASMAS bench top system), keeping the temperature and RH (see Sec. 1.1).

### 2.2 Preparation of Liquid Phantom with Optical Properties of Lung Tissue

The optical properties of lung tissue at 760 nm were assigned by filling the capillaries with liquid phantom. To produce 100 ml phantom, 8.5 ml of intralipid (SIGMA, Lot # MKCL8461) was mixed with 1.5 ml of stock ink-water solution (1% Winsor & Newton® black India ink diluted in 99 % distilled water) and 90 ml distilled water. The *g*-factor was defined by the scattering of intralipid g=0.6,^23^ and the optical properties were determined using the fiber optic probe system based on diffuse reflectance and diffuse spectroscopy from Ref. 24.

The capillary tubes used to mimic lung absorption and scattering were filled using capillary action. In some cases, air bubbles prevented the rising of the liquid in the tube, which was solved by gently tapping on the external side of the tube. The top end of the tubes was then sealed with starch-based modeling clay (Play-Doh) to prevent leakage during measurements.

The liquid phantom and air-filled capillaries were then randomly placed by hand into the holder to make up the capillary arrays for absorption measurements. The probed air volume was changed by varying the ratio of air-filled capillaries in the array (Sec. 3.2).

### 2.3 Chamber for Controlled Environment, Mimicking Lung Relative Humidity, and Temperature

**Fig. 2:**
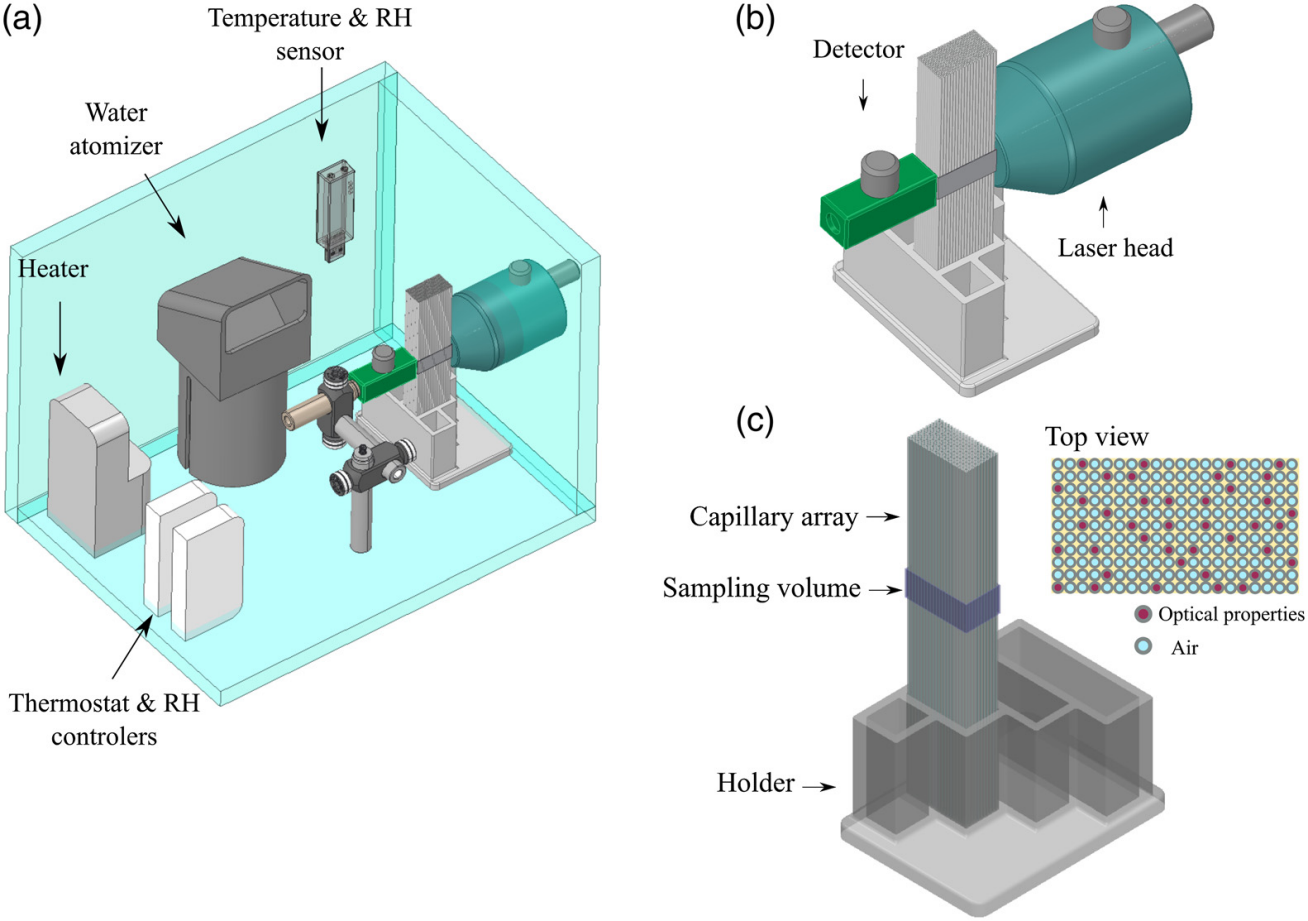
(a) Diagram of the experimental set up designed to study the variations in GASMAS signal for different gas volumes in the lung tissue phantom. (b) Zoom in of source and detector placement in transmitance geometry. (c) The capillary array placed in the 3D printed holder, the sampling volume was defined by the size of light source and detector and the length of the capillary array.

Figure 2 shows the diagram of all the components used in the lung model. An enclosure with temperature and RH control was built using 12-mm thick acrylic sheets (Blarney Trading. Cork, Ireland). The overall dimensions of the enclosure were 0.28×0.22×0.20  m (L×H×W). The temperature was controlled using a heater (50W, STEGO, Radionics Ireland) and a thermostat (Schneider Electric, Radionics Ireland) set to 37°C. To help maintain the temperature in the enclosure, the outer walls were covered with 0.3-m thick panels of expanded polystyrene foam. The RH was controlled via a generic fish tank water atomizer coupled to a humidity meter (Schneider Electric, Radionics Ireland) set to 90% RH (maximum range in the controller settings). The atomizer was placed inside a 200-ml Pyrex beaker filled with 50 ml of tap water. To prevent large drops of water being ejected from the beaker by the atomizer, an in-house 3D-printed catch lid was installed into the beaker. Both temperature and RH were monitored using a digital sensor (TSP01, ThorLabs). An opening was drilled in one of the walls of the enclosure for the dual laser head of the MicroLab Dual $O_{2}/H_{2}O$ (see Sec. 3.1). The detector was mounted inside the enclosure on adjustable posts (RA90/M and TR75V/M, ThorLabs) and positioned directly in front of the source, to measure the gas content in the capillary array.

The capillary array was placed between the source and detector in transmission geometry. The long walls of the capillary array were masked with a black film, and the contact surfaces between the capillaries and the system source and detector were daubed with ultrasound gel, to prevent light absorption by the gas outside the capillary tubes.

## 3 GASMAS Measurements

### 3.1 MicroLab Dual O_2_/H_2_O

The GASMAS bench top system used in this study was MicroLab Dual $O_{2}/H_{2}O$ (GASPOROX AB). The system was equipped with a dual diode laser source operating at 760 and 820 nm as well as a photodetector with $1\times 10^{-4}\;m^{2}$ area. The values of temperature (taken with the Thorlabs probe) and room pressure (1.007 Atm) were input to the system prior to data acquisition.

One wall of the capillary array was illuminated using the 760- and 820-nm lasers. During the illumination, each laser scanned across the absorption lines of $O_{2}$ (760 nm) and $H_{2}O$ (820 nm). The scattered light was collected by the detector placed on the opposite side of capillary array. The system employed wave modulation spectroscopy (WMS) to increase the detection sensitivity of $O_{2}$ and $H_{2}O$. To use this method, the detection band of each gas was modulated to higher frequencies (in the kilohertz range). Simultaneously, the laser wavelength was scanned across the gas absorption line, generating high harmonics of the absorption signal and suppressing the background noise. The characteristic output of this instrument was the amplitude signal obtained by WMS, which was used to detect the proportional light intensity dip for each wavelength.

### 3.2 Air Volumes to Test in the Pulmonary Phantom Model

The sampling air volume was defined by the dimensions of the diffuse light source and detector (0.01×0.005  m and 0.01×0.01  m, respectively) and the length of the capillary array (0.025 m) as shown in Fig. 2(c). The air volume between the capillaries ($6.89\times 10^{-7}\;m^{3}$), constant to all measurements, was calculated by subtracting the total volume occupied by 229 capillaries ($2.81\times 10^{-6}\;m^{3}$) from a cube with the dimensions of the analyzed volume ($3.5\times 10^{-6}\;m^{3}$).

An alveolar unit holds $∼1.41\times 10^{-8}\;m^{3}$ of air, assuming a spherical geometry. The air content of a single capillary in our model contained $1.96\times 10^{-9}\;m^{3}$ of air. The model could hold a maximum of $3.5\times 10^{-6}\;m^{3}$ of air within the sampling volume, analogous to a group of alveolar sacks with at least 250  inflated alveoli. The radius of the capillaries used in this study was in agreement with the physiological radius of an alveolar unit. Despite the cylindrical geometry, the air volumes were comparable with those of pulmonary tissue and the lung model made possible to recreate progressive changes in volume during respiration in a pulmonary section.

The test set 1 started with all the capillaries empty, and the absorption signal was measured 10 times. For each acquisition, the MicroLab Dual $O_{2}/H_{2}O$ system averaged the absorption imprints from $O_{2}$ and $H_{2}O$ over 10 s. The air content between source and detector was reduced by replacing (and allocating randomly by hand) 20 empty capillaries with those filled with liquid phantom, followed by the respective data acquisition. This process was repeated 10 times until a total of 200 capillaries were filled with lung tissue phantom and remaining 29 filled with air, as shown in Table 1. In the test set 2, the inverse process was done. It started with all capillaries filled with optical properties and a progressive increment in air volume of 20 capillaries at a time followed by the data acquisition. The process was repeated until a total of 200 empty and 29 capillaries filled with liquid phantom were placed in the holder.

**Table 1:** Air volumes in the capillary lung model conformed by 229 capillaries in total and a constant air space of $6.89\times 10^{-7}\;m^{3}$.

Test set 1: Air volume progressively reduced		Test set 2: Air volume progressively increased	
Capillaries filled with lung liquid phantom	Volume of air tested ($\times 10^{-6}\;m^{3}$)	Capillaries filled with air	Volume of air tested ($\times 10^{-6}{\;m}^{3}$)
0	1.14	0	0.69
20	1.10	20	0.73
40	1.06	40	0.77
60	1.02	60	0.81
80	0.99	80	0.85
100	0.94	100	0.89
120	0.90	120	0.93
140	0.86	140	0.97
160	0.83	160	1.00
180	0.77	180	1.04
200	0.75	200	1.08

## 4 Results and Discussion

The path length in GASMAS measurements correspond to the effective path that light travels inside the gas cavities where the absorption dominates over scattering. The surrounding bulk material (in this case the liquid phantom) attenuates the amount of light reaching the detector. The augmentation of liquid phantom-filled capillaries within the measurement sets increased the light diffusion within the phantom and at the same time reduced the number of gas cavities. The absorption path length in highly diffuse materials, such as biological tissue, is unknown. In this study, the gas concentration of $H_{2}O$ was known, enabling the retrieval of the gas absorption path length (see Sec. 1.2), which was assumed to be the same for molecular $O_{2}$.

**Fig. 3:**
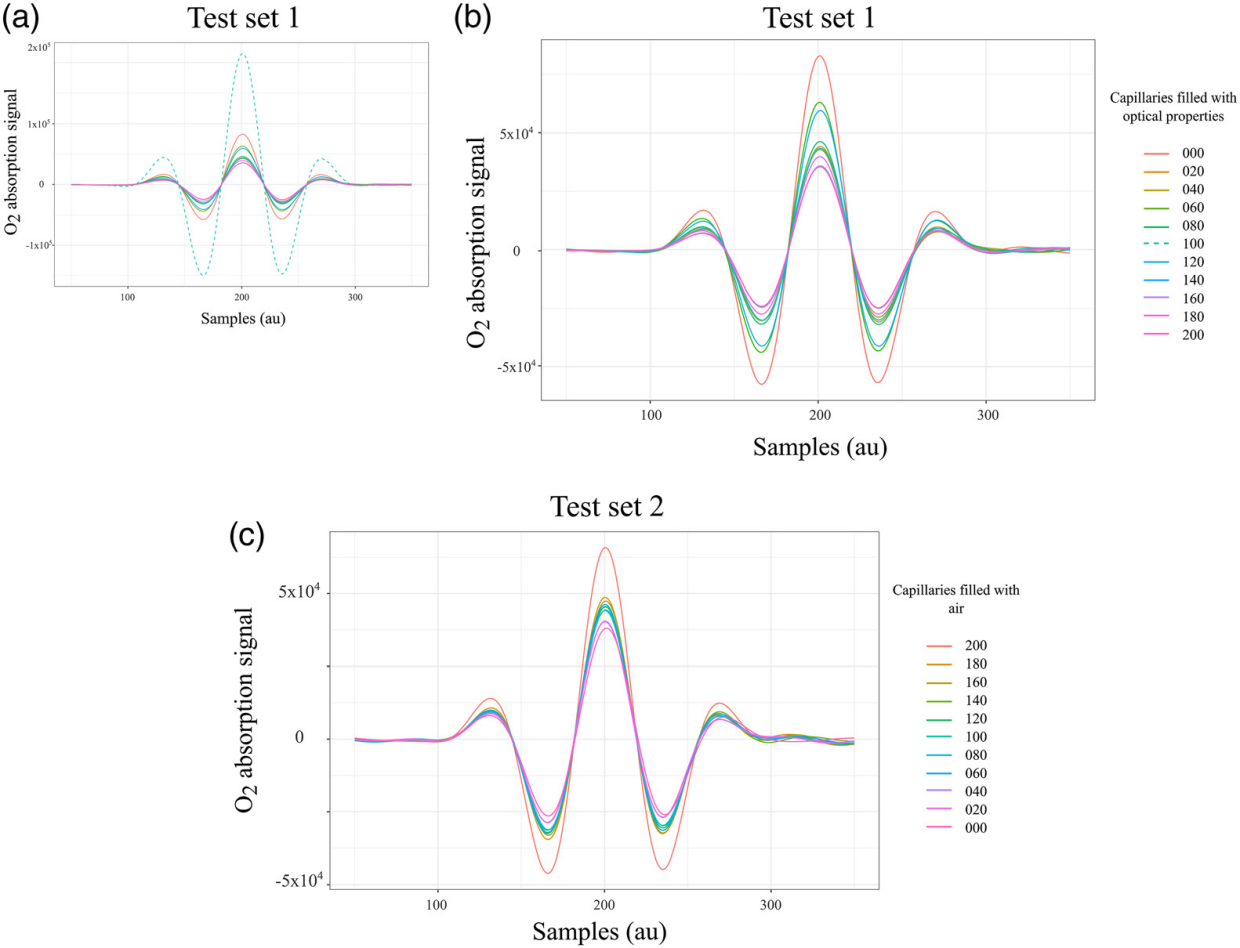
WMS amplitude signals that correspond to the absorption of molecular oxygen tested in the lung model in transmittance geometry. (a) Complete test set 1, the acquisition started with all capillaries filled with air followed by a progressive reduction of air volume. The dashed line exhibits higher absorption compared with the absorption of the maximum sampling volume, therefore these data were removed in (b) for the analysis. (c) Signals from test set 2, the acquisition started with all capillaries filled with optical properties followed by a progressive increase of air volume.

The absorption signals acquired for the test set 1 are shown in Fig. 3(a), the dashed line corresponds to the capillary array with 100 units filled with optical phantom and 129 with air. The observed level of absorption resembled a condition where the air volume was larger than the maximum capacity of the sampling volume. For this reason, the measurements from that configuration were omitted from data analysis as shown in Fig. 3(b). The absorption signals from the second test set are plotted in Fig. 3(c).

According to Eq. (1), the absorptivity increases proportionally to the gas absorption path length, this is corroborated by the signals plotted in Figs. 3(b) and 3(c), which exhibit highest absorption for the arrays with maximum air volume, followed by a decrease in the absorption signal as the capillaries filled with optical phantom reduced the air volume. The $O_{2}$ concentration calculated during the first test set was 26.3% (SD=2.3%) compared with 25.4% (SD=2.9%) from the second test set.

**Fig. 4:**
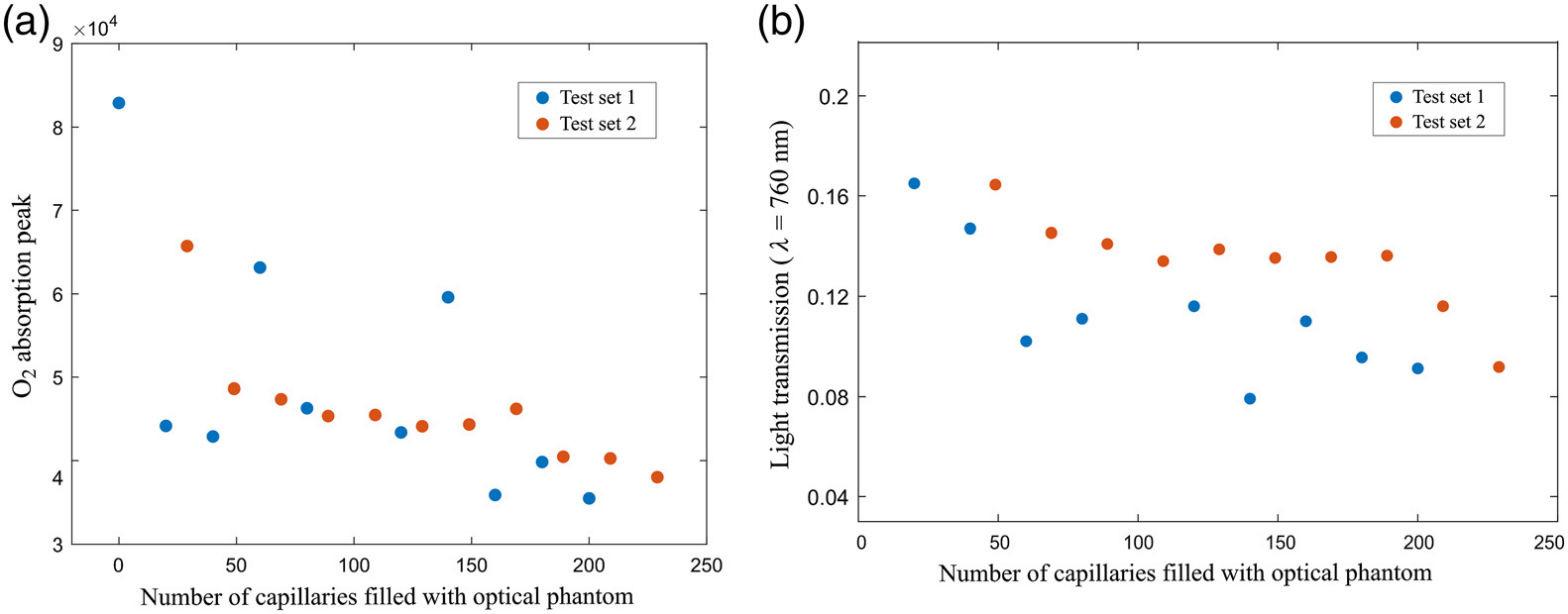
(a) Peak values of the WMS absorption signals of molecular oxygen as a function of the number of capillaries filled with liquid phantom; and (b) light transmission values for the capillary arrays with optical phantom.

The peak values of the absorption signals for sets 1 and 2 are plotted as a function of the number of capillaries filled with optical phantom in Fig. 4(a). As previously stated, the observed decreasing absorption peak trend was in agreement with decreasing volume of oxygen versus the gas absorption path lengths. The light transmission for the configurations that included a number of capillaries filled with liquid phantom was plotted in Fig. 4(b). The light transmission decreased as the population of capillaries with optical properties grew. It is likely that this reduction in measured intensity also contributed to an error in absorption values. In this model, air volume and alveolar geometry were well defined, whereas in the human lung the air content and the geometry of the alveolar sacks change. A detailed understanding of variations in the light transmission could be linked to physiological values of alveolar inflation or deflation during respiration. The increment (or reduction) of air volume in the model was made in steps of 20 capillaries for each data acquisition set. The test set 1 exhibited higher variation in the absorption signals compared to the test set 2. This difference was likely related to the stability of temperature and RH during data acquisition. GASMAS requires a high humidity to estimate accurately the $H_{2}O$ concentration. Ideally, RH should be ~100%, but in this experiment, the RH was oscillating in the range of 90% to 94% due to condensation, and this variation also induced temperature changes between 36°C and 39°C during the data acquisition. Such fluctuations are not present in the human lung, because the body regulates the temperature to 37°C and the cooling effect associated with water condensation is nonexistent. The outlier data from test set 1 in Fig. 4(a) corresponded to 60 and 140 capillaries filled with phantom and exhibited poor light transmission as shown in Fig. 4(b).

This study was done using steady volumes for each data acquisition. A new version of the lung model with time dependence could be built, connecting the capillaries to a pumping system, to recreate tidal respiratory volumes.

The latest development in light sources and detectors could enable the implementation of GASMAS into pulmonary endoscopy, in a similar way as optical coherence tomography has become a complementary tool in bronchoscopy procedures.^25^ GASMAS technology could be integrated into pulmonary endoscopes using optical fiber-based coupling. This has the potential to improve the patient-specific information content that will help the clinicians with lung obstruction assessment and track the patient’s response to bronchodilators or hyperinflation-reducing therapies. The implementation of GASMAS technology in the clinic created the capability to locally assess the inflation of lung tissue, which is not possible with the existing diagnostic procedures such as spirometry and plethysmography.

## 5 Conclusion

A phantom with the optical properties and the structure of tissue from the respiratory zone has been modeled using a simplified capillary array immerse in a controlled environment chamber maintaining temperature and RH in the range of the human body.

The lung model enabled study of the changes in the GASMAS signals generated by the MicroLab Dual $O_{2}/H_{2}O$ system in transmittance geometry. The feasibility of sensing volume changes with GASMAS technique has been proven, stating a new possible application of GASMAS technology in respiratory treatment and diagnostics. This is a significant contribution since, to the best of our knowledge, previous studies addressing the clinical translation of GASMAS into respiratory healthcare reported on sensing the absorption imprint of $H_{2}O$ and $O_{2}$ to quantify gas concentrations.

## Code, Data, and Materials Availability

The code and data used for this study are available upon request.
